# The function, regulation and therapeutic implications of the tumor suppressor protein, PML

**DOI:** 10.1186/s13578-015-0051-9

**Published:** 2015-11-04

**Authors:** Dongyin Guan, Hung-Ying Kao

**Affiliations:** Department of Biochemistry, School of Medicine, Case Western Reserve University, and Comprehensive Cancer Center of Case Western Reserve University, Cleveland, 10900 Euclid Avenue, Cleveland, OH 44106 USA

**Keywords:** PML, PML nuclear bodies, Tumor suppressor protein, Therapy

## Abstract

The tumor suppressor protein, promyelocytic leukemia protein (PML), was originally identified in acute promyelocytic leukemia due to a chromosomal translocation between chromosomes 15 and 17. PML is the core component of subnuclear structures called PML nuclear bodies (PML-NBs), which are disrupted in acute promyelocytic leukemia cells. PML plays important roles in cell cycle regulation, survival and apoptosis, and inactivation or down-regulation of PML is frequently found in cancer cells. More than 120 proteins have been experimentally identified to physically associate with PML, and most of them either transiently or constitutively co-localize with PML-NBs. These interactions are associated with many cellular processes, including cell cycle arrest, apoptosis, senescence, transcriptional regulation, DNA repair and intermediary metabolism. Importantly, PML inactivation in cancer cells can occur at the transcriptional-, translational- or post-translational- levels. However, only a few somatic mutations have been found in cancer cells. A better understanding of its regulation and its role in tumor suppression will provide potential therapeutic opportunities. In this review, we discuss the role of PML in multiple tumor suppression pathways and summarize the players and stimuli that control PML protein expression or subcellular distribution.

## Background

**Fig. 1:**
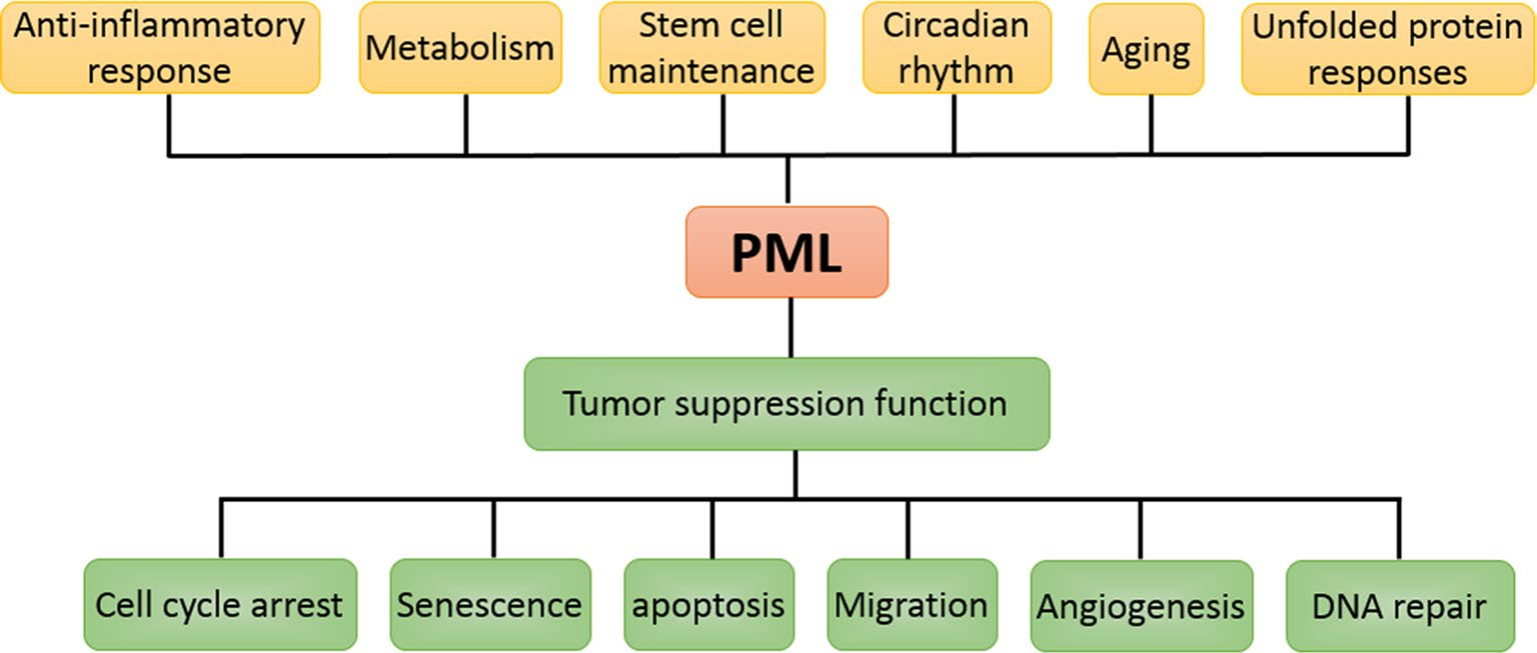
Summary of PML functions in diseases. PML plays pivotal roles in the indicated conditions including anti-inflammatory responses, metabolism, stem cell maintenance, circadian rhythms, aging and unfolded protein responses. PML protein exerts its tumor suppressive function by regulating the cell cycle, apoptosis, senescence, migration, angiogenesis, and DNA repair pathways

In the early 1990s, groundbreaking discoveries in PML research attracted the attention of cancer researchers. The first breakthrough was the mapping of the breakpoint of a reciprocal chromosomal translocation between chromosomes 15 and 17 involved in acute promyelocytic leukemia (APL) [1, 2]. The promyelocytic leukemia gene (PML also known as MYL, RNF71, TRIM19 and PP8675) was first described as a fusion partner of the retinoic acid receptor alpha (RARα), generating the oncogenic protein (PML-RARα), which is present in >98% of APL cases [3]. Twenty-five years of intense study on the PML protein from many laboratories has led to the conclusion that PML is a multi-faceted protein that plays pivotal roles in cellular events under physiological and pathological conditions [4–6]. These include its role in tumor suppression, anti-viral and anti-bacterial responses, inflammatory responses, metabolism, aging, circadian rhythm and unfolded protein responses (Fig. 1) [6–9]. Understanding the mechanism by which PML participates in these processes will facilitate development of therapeutic strategies for the treatment of PML-related diseases. Here, we review the literature and highlight recent progress with a focus on our current understanding of the role of PML in tumor suppression.

## PML and PML-nuclear bodies (NBs)

**Fig. 2:**
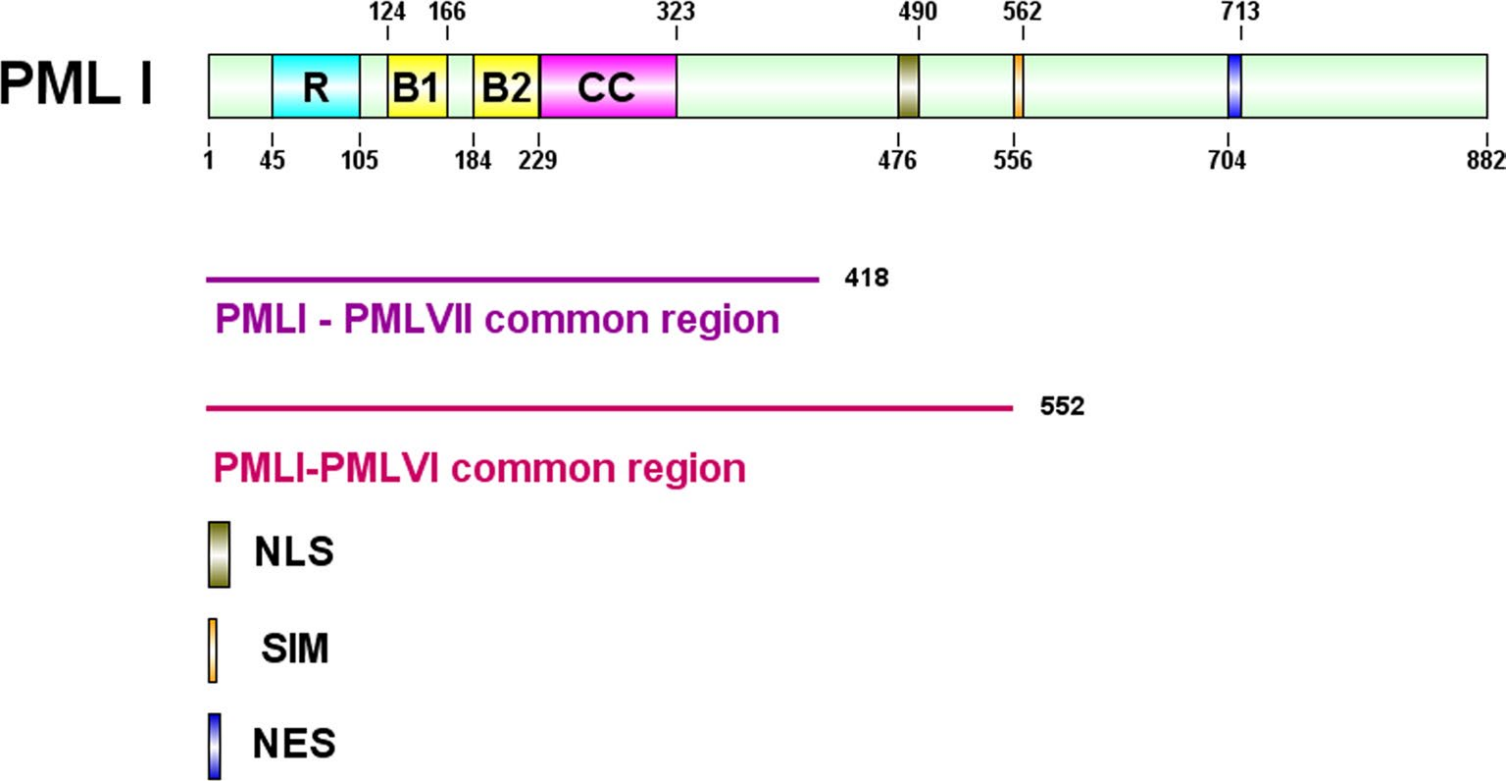
A schematic diagram of PML functional domains. All PML isoforms share the same N-terminal 418 amino acids, which contain RING (*R*), B-Box1 (*B1*), B-Box1 (*B2*) and coiled coil (*CC*) domains. Nuclear PML isoforms share the same N-terminal 552 amino acids, which in addition to RBCC, contains a nuclear localization signal (*NLS*) and a SUMO-interacting motif (*SIM*) (66) present in PML isoforms I–V. Only PMLI contains a putative nuclear export signal (*NES*) at its C-terminus

The *Pml* gene contains nine exons and spans approximately 53 kb in the genome. Due to alternative splicing of its C-terminal exons, six nuclear and one cytoplasmic isoform have been experimentally verified. PML I is the longest isoform and contains 882 amino acids, while the shortest isoform, PML VII, has 435 amino acids [4, 10]. The N-terminal 418 amino acids are common to all isoforms and harbor several structurally conserved domains that include a Really Interesting New Gene (RING) finger domain (R), two cysteine/histidine-rich B-Box domains (B1 and B2) and an α-helical coiled-coil domain (CC) (Fig. 2). Collectively, these domains are referred as the RBCC domain or the tripartite motif (TRIM) [11, 12]. The distinct C-terminal sequences of PML isoforms suggest that there are isoform-specific functions. For example, in response to type I interferon (IFN), PML II is specifically required for the induction of IFN-stimulated genes transcription via formation of transcriptional complexes with NF-κB, STAT1 and CBP [13]. PML II and V can form PML NB-independent of the N terminal, RBCC domain [14]. A detailed review of isoform-specific functions of PML was recently published by Nisole et al [10].

PML has been shown to be enriched in nuclear punctate structures that are interspersed between chromatin [15]. These structures have been variably named PML nuclear bodies (PML-NBs), Kremer bodies, ND10 (nuclear domain 10) or POD (PML oncogenic domains) [15]. PML-NBs are heterogeneous and dynamic structures. The RBCC domain is essential for PML-NB formation [16, 17]. The size of PML-NBs ranges from 0.1 to 1.0 µm and typically there are 5–30 bodies per nucleus, depending on the cell types, phase of cell cycle, stress and nutritional condition [18]. Loss of PML results in a loss of PML-NBs, indicating that PML protein is an essential component of PML-NBs [5, 19, 20]. Two models of how PML NB formation is initiated have been proposed. Based on the identification of a C-terminal SUMO interacting motif (SIM) and the sequence requirement in PML for co-localization with GFP-SUMO1, it was proposed that the nucleation of PML NBs depends on PML sumoylation and non-covalent interactions of SUMOylated PML and PML SIM [16]. However, PML VI (NP_150247.2), which does not have the SIM, a mutant missing the SIM, or PML 3 KR, which has lost all three SUMOylation site, are still capable of forming nuclear bodies and form PML polymers [16]. An alternative model suggests that PML NB formation is a two-step process. In the first step, PML NB formation relies solely on reactive oxygen species (ROS)-induced PML oxidation, resulting in covalent bonds between PML monomers. Subsequently, a polarized SIM-SUMO-dependent mechanism recruits sumoylated or SIM-containing partner proteins, such as DAXX, followed by an increase in PML NB formation [21]. The later model partly explains why PML 3 KR and SIM-deficient isoforms still form nuclear aggregates.

**Fig. 3:**
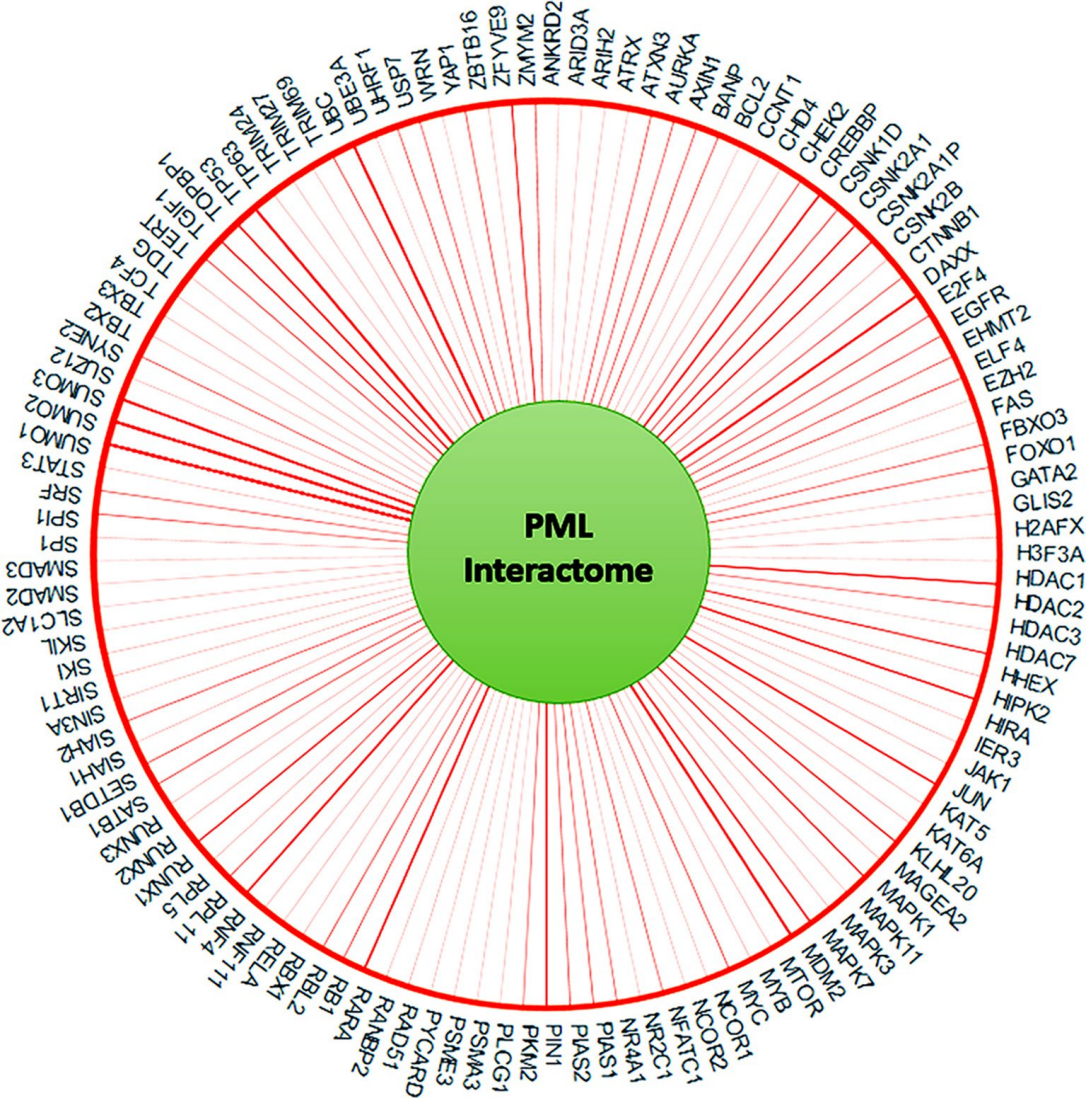
PML interactome. Based on the data from BIOGRID (http://www.thebiogrid.org/), 120 proteins transiently or constitutively physically interact with PML. This has been demonstrated by affinity capture followed by Western blotting experiments. The PML-associating proteins identified by high-throughput methods are not included. The *thicker* *of the line* the more publications support the association

It has been estimated that PML functionally interacts with more than 160 proteins directly or indirectly [22]. Based on information in BIOGRID (http://www.thebiogrid.org/), 120 proteins physically interact with PML, either transiently or constitutively, as demonstrated by affinity capture experiments followed by Western blotting (Fig. 3). These interactions suggest the possibility of mutual regulation between PML and its interacting partners [23–25].

## The role of nuclear PML and PML-NBs in tumor suppression

Five years after the discovery of the *Pml* gene, the tumor suppressive activity of PML was demonstrated in several cancer types including breast, lung, colorectal, prostate and bladder cancer [26–30]. Overexpression of PML inhibits cell proliferation and leads to cell cycle arrest, senescence and apoptosis whereas *Pml* knockout cells exhibit increased proliferation and resistance to UV and cytokine-induced apoptosis [31–33]. Moreover, *Pml* knockout mice demonstrated elevated spontaneous and chemically-induced tumorigenesis [32]. These data suggest that PML is a tumor suppressor. PML-NBs are thought to function as nuclear storage sites that accumulate or sequester proteins in order to release these proteins when required [34]. Recent studies indicated that PML-NBs mediate protein-protein interactions and functions as a platform that promotes protein post-translational modification, for example, SUMOylaiton, acetylation, ubiquitination and phosphorylation [35].

**Fig. 4:**
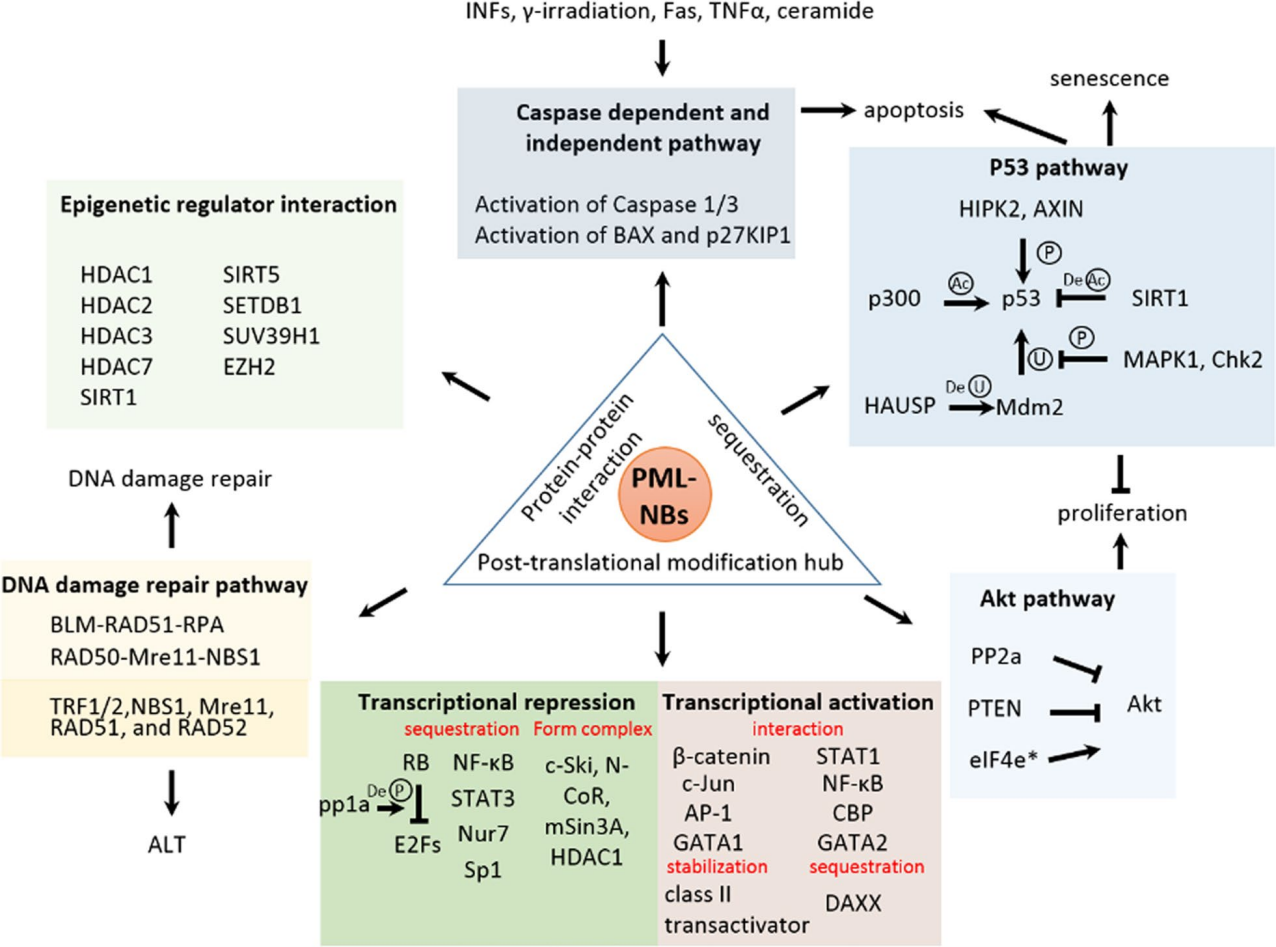
PML NB-mediated tumor suppression pathways. PML NBs repress protein function by sequestration, mediating protein–protein interaction, or acting as a post-translational modification hub to regulate diverse tumor suppressor pathways

Several distinct mechanisms underlying PML-mediated tumor suppression activity have been reported (Fig. 4): [1] PML sequesters proteins in PML-NBs to repress their functions, [2] PML recruits proteins to PML-NBs or mediates protein-protein interaction to activate their function, [3] PML-NBs serve a post-translational modification hub to regulate protein activity and function, [4] PML facilitates targeting of transcription factors and co-regulators to specific region of genome to control gene expression, [5] PML and PML-NBs are a part of complexes that regulate DNA damage repair and [6] PML mediates alternative lengthening of telomeres (ALT) to maintain genome integrity. These mechanisms influence important cellular pathways such as apoptosis, p53 stability, Akt activity and gene regulation.

### Caspase 3-dependent and -independent pathways in apoptosis

The activation of caspase 3 is a key event in apoptosis and is vital for the inhibition of cancer cell growth [36]. PML induces caspase 3 activation and mediates multiple apoptotic pathways in response to various stimuli, including γ-irradiation, tumor necrosis factor α (TNFα), Fas, type I and II interferon (INFs), and ceramide [37, 38]. The lethal effects of γ-irradiation and anti-Fas antibody are attenuated in *Pml* knockout mice and cells [38, 39], indicating that *Pml*-mediated activation of caspase 3 is essential for apoptosis. However, PML can also recruit BAX and p27KIP1 to PML-NBs and can mediate apoptosis independently of caspase 3 activation [39]. In summary, PML mediates apoptosis via both caspase 3-dependent and -independent pathways.

### Regulation of p53

The tumor suppressor p53 is an extensively studied gene that is important for many aspects of tumor biology [40]. PML is a critical regulator of p53 activity and p53-mediated cellular processes, such as apoptosis, cell cycle arrest, DNA repair and senescence. In response to cellular stress and DNA damage, PML enhances p53 protein stability by sequestering Mdm2 in NBs [41–43]. Mdm2 is a major cellular p53 E3 ubiquitin ligase that destabilizes p53. The activated big MAP kinase 1 (BMK1) interacts with PML and disrupts its association with Mdm2, thereby destabilizing p53 [44]. Furthermore, DNA damage promotes the recruitment of the DNA damage-induced kinase Chk2 to PML-NBs where it phosphorylates p53 at serine 20, thereby blocking the interaction between Mdm2 and p53, and subsequently alleviating p53 degradation [45]. In response to DNA damage or ultraviolet-induced apoptosis, the acetyltransferases CBP/p300 and the homeodomain-interacting protein kinase-2 (HIPK2) along with the tumor suppressor AXIN are recruited to PML NBs where CBP/p300 and HIPK2 acetylate p53 at K382 and phosphorylate it at Ser46, respectively [46–49]. Both of these modifications activate p53 transcriptional activity and induce cell apoptosis or senescence [18, 49]. By contrast, the deacetylase SIRT1 is also recruited to PML-NBs upon overexpression of PML or activation of oncogenic Ras (Ha-ras V12) and deacetylation of p53 by SIRT1 represses p53 transcriptional activity [50]. Thus, p53 can be stabilized or destabilized in PML-NB depending on the composition of the NBs. This may be cell type-specific and dependent on the conditions used in these studies. In sum, PML is capable of regulating p53 protein abundance and activity by multiple mechanisms that include sequestration of Mdm2-dependent PTM of p53 and SIRT1- dependent deacetylation of p53.

### Akt pathway

Activation of Akt results in phosphorylation of numerous substrates, which regulate metabolism, survival, migration and cell cycle progression [51]. PML inhibits Akt activation by sequestering Akt and recruiting protein phosphatase 2a (PP2a) to PML-NBs [52]. In PML-NBs PP2a dephosphorylates Akt and inhibits its kinase activity [52]. Furthermore, PML can suppress Akt activity via the eIF4E-NBS1-PI3 K-Akt axis [53]. PML directly interacts with and negatively regulates elF4E activity in PML NBs, thereby reducing eIF4E-dependent mRNA export, including mRNA for *NBS1*, an upstream activator of the phosphoinositide-3 kinase-Akt pathway [53]. PML also positively regulates PTEN (phosphatase and tensin homolog), a suppressor of PI3 K/Akt activation. Monoubiquitination of PTEN is required for its nuclear localization and tumor suppressor activity and deubiquitination by the deubiquitinase HAUSP blocks PTEN nuclear localization [54]. Inactivation or loss of PML results in a decrease in nuclear PTEN [55]. In PC3 prostate cancer cells, overexpression of PML opposes HAUSP deubiquitination activity. PML binds to and inhibits the death domain associated protein DAXX, which stabilizes HAUSP [55]. A recent study reported that cytoplasmic PML is also essential for Akt- and PP2a-dependent activation of 1,4,5-triphosphate receptor (IP [3] R) phosphorylation, which triggers calcium release from the endoplasmic reticulum to initiate apoptosis [56]. In summary, PML regulates cell proliferation and survival by inhibiting Akt kinase activity through PP2a, eIF4E and HAUSP.

### Potentiation of Rb activity

The retinoblastoma protein (RB) is a potent tumor suppressor through its inhibitory effect on E2F transcription factors hosphorylation of RB (pRB) blocks its interaction with E2F and promotes cell cycle progression. PML-NBs recruit protein phosphatase 1α (pp1a), which dephosphorylates RB, thereby promoting the interaction between RB and E2Fs and repressing E2F-driven transcription and cell cycle progression [57, 58]. Oncogenic Ras induces PML protein expression in mouse embryonic fibroblasts (MEFs), which results in colocalization of RB to PML NBs, and hypophosphorylation of RB with subsequent cell senescence [59].

### Transcriptional regulation by PML

PML-NBs can sequester the NF-κB subunit RelA/p65, and inhibit its transcriptional activity in TNFα-induced apoptosis [60]. A20 is a NF-κB target gene that inhibits TNFα-induced apoptosis in a negative feedback fashion. PML represses NF-κB-mediated *A20* transcription by preventing NF-κB from binding to the *A20* promoter [61]. PML can also sequester Sp1 and Nur77 to NBs and disrupt their binding to target promoters [62, 63]. PML interacts and inhibits STAT3 by inhibiting STAT3 DNA binding activity [64]. PML also forms complexes with multiple corepressors (c-Ski, N-CoR, and mSin3A) and histone deacetylase 1 (HDAC1), which are required for the tumor suppressor Mad to mediate its transcriptional repression [65]. Together, these studies support a model in which PML represses gene transcription by sequestering transcription factors to PML-NBs or by associating with transcriptionally repressive complexes.

In contract, several mechanisms have been proposed by which PML positively regulates transcription. PML and PML-NBs recruit DAXX, which also functions as a transcriptional co-repressor, thereby transcriptionally derepressing DAXX target genes, such as Pax3 [66] and GRα target genes [67–69]. Such regulation depends on Sumo1 conjugation of PML and a SIM (sumo interacting motif) in DAXX. We have previously reported that in response to TNFα stimulation, PML NBs sequester HDAC7, reducing its association with the *MMP*-*10* promoter, thereby inducing *MMP*-*10* expression [70]. In addition, PML blocks degradation of the class II transactivator (CIITA), thereby stabilizing the protein, and promotes the expression of its target genes that include the class II major histocompatibility complex [71]. The PML II isoform associates with transcription factors NF-κB, STAT1 and CREB-binding protein (CBP) to facilitate transactivation complex formation and activate interferon beta (INFβ) and interferon-responsive gene (IRG) expression [13]. However, it is not known whether PML II is present at these promoters. PML, p300 and β-catenin form complexes and activate the transcription of a subset of β-catenin responsive genes that include *ARF* and Siamois [72]. Interestingly, PML collaborates with the known oncoprotein c-Fos and enhances AP-1 transcriptional activity in a transient transfection reporter assay [73] and is essential for c-Jun DNA binding and transcriptional activation in response to UV irradiation [74]. PML is also required for all-trans retinoic acid (AT-RA)-induced transactivation of the p21^WAF1/CIP1^ gene [32]. Moreover, PML physically associates with GATA1 and GATA2, the master transcriptional factors of hematopoietic stem cell development, facilitating their transcriptional activities [75, 76]. In summary, PML activates gene transcription through sequestration of transcriptional co-repressors, stabilization or post-translational modification of transcriptional factors, and possibly other mechanisms yet to be elucidated.

### The role of PML in DNA damage repair

Recently it has been suggested that PML and PML-NBs play a critical role in DNA damage repair and ALT [77–79]. ALT is an alternative mechanism of telomere maintenance in immortalized human cells and cancer cells that is telomerase-independent [80]. In ALT cells, PML co-localizes with telomeric DNA, the telomere-binding proteins TRF1 and TRF2 as well as proteins involved in DNA synthesis and recombination, such as NBS1, Mre11, Rad51 and Rad52 [78, 79]. By binding these proteins, PML and PML-NBs play a role in DNA damage responses, which are is important for the maintenance of genomic stability and integrity in ALT cells [81].

PML also co-localizes, associates with and stabilizes the DNA damage response protein, TopBP1 after ionizing radiation (IR) [82]. Upon the induction of double strand breaks (DSBs), NBS1, ATM, Chk2 and ATR facilitate biogenesis of PML-NBs [83]. The 3′ → 5′ DNA helicase, BLM, is an important regulator of the maintenance of genomic stability and has also been shown to reside in PML-NBs [84]. Interestingly, loss of BLM or PML results in increased numbers of sister-chromatid exchanges (SCE). BLM, RAD51 and replication protein (RP)-A assemble in PML-NBs during late S/G2 phase in undamaged cells and again after DNA damage [85]. The RAD50-Mre11-NBS1 complex is implicated in the maintenance of telomere length in the absence of telomerase and plays a role in repair of DSBs, including homologous and non-homologous recombination repair (NJEM) [86]. Following IR treatment, the RAD50-Mre11-NBS1 complex is co-localized in PML-NBs at sites of DSBs, suggesting a role of PML in repair of DSBs [87, 88]. In summary, PML has multiple roles in both DNA damage repair and maintenance of genomic stability.

## Janus-faced role of cytoplasmic PML in tumorigenesis

Cytoplasmic PML has been reported to have both oncogenic and tumor suppressive functions in different biological contexts. A cytoplasmic isoform of *PML* that contains exons 1–4, 6 and 7 and part of exon 9, was identified in plasmacytoma J558 cells [89]. This isoform of PML contributes to MHC class I antigen presentation, and enables tumors to evade the immune defense of its host [89]. In APL cells, the PML-RARα fusion protein can be cleaved after V420 or V432 of the PML protein by neutrophil elastase and form a truncated PML protein that does not have the NLS and localizes in the cytoplasm [90]. Mutations in PML are not common but cytoplasmic PML can also result from mutations. A small deletion (1272delAG) and a splice site mutation (IVS3-1G → A) in the *PML* gene have been identified in aggressive from of APL. The mutant PMLs generated are truncated and do not have a nuclear localization signal (NLS) [91]. They localize in the cytoplasm due to a premature stop codon before the NLS. Cells from the APL patients are resistant to retinoic acid treatments and have reduced levels of apoptosis and increased proliferation [90–93]. In addition, the previously described truncated PML mutant derived from APL mutations [91] can sequester nuclear PML in the cytoplasm through dimerization and inhibits p53 tumor suppressive functions [94]. Additionally, increased expression and cytoplasmic localization of PML was observed in a hepatocellular carcinoma [95, 96]. However it was unclear whether the PML in this tissue contained mutations. Therefore, whether cytoplasmic wild-type PML promotes tumorigenesis is still debatable.

Emerging evidence suggests that cytoplasmic PML can also have tumor suppressor functions. The M2 type pyruvate kinase (PKM2) is overexpressed in many cancers [97]. A PML mutant, which harbors an NLS mutation and is constitutively cytoplasmic, interacts with and inhibits PKM2 activity and lactose production [98]. The transforming growth factor beta (TGFβ) can promote or suppresses tumorigenesis, depending on the cellular context [99]. Lin et al. reported TGFβ treatment for 24 h specifically induces a cytoplasmic PML isoform, which contains exons 1–3, 7a, 8a and 8b and lacks the NLS. This cytoplasmic PML isoform facilitates the assembly of the TβRI/TβRII/SARA/Smad2/3 complex in endosomes and is required for Smad2/3-dependent transcription. Such transcription is critical for TGFβ-mediated inhibition of cell proliferation, apoptosis and cell senescence [100]. Additionally, overexpression of the homeodomain protein TGIF results in nuclear retention of such cytoplasmic PML and blocks TGFβ signaling [101]. Together, these reports conclude that cytoplasmic PML regulates the TGFβ pathway to promote its tumor suppressor activity.

In MEFs, a fraction of PML localizes to the endoplasmic reticulum and to mitochondria-associated membranes (MAM) [56]. At these sites, PML forms a complex with IP [3] R, Akt and PP2a. Overexpression of a fusion protein containing the entire PML protein that was targeted to the outer surface of the ER in MEFs promotes apoptosis by stimulating calcium release. In *PML*^−*/*−^ MEFs, Akt-dependent phosphorylation of IP [3] R is enhanced and calcium release from ER is decreased, thereby impairing the apoptosis response to H_2_O_2_ or menadione [56]. These findings suggest that cytoplasmic PML possesses tumor suppressive activity.

## Regulation of PML expression and therapeutic opportunities

Inactivation of PML in cancer cells occurs through multiple mechanisms [26, 102]. However, only few somatic mutations have been reported so other mechanisms must be involved [26, 102]. Studies have indicated that effects on PML accumulation occur are at the transcriptional and post-translational levels. Epigenetic regulation of PML expression and alternative splicing of *PML* mRNA are less well studied [35]. In many types of cancers, down-regulation of PML protein, but not its mRNA, is observed. Thus, post-transcriptional regulatory mechanisms are involved [26–29]. This observation provides therapeutic opportunities to target cancer cells with the goal of restoring PML protein expression by altering PML translation, localization or post-translational modification.

### Transcriptional and translational regulation

Several reports have suggested that inflammation-associated cytokines enhance *PML* transcription. The *PML* promoter contains an IFNα/β stimulated response element and an IFNγ binding site [103, 104]. Interferons (INFs) have been shown to induce senescence [105], a key anti-cancer mechanism. IFNs induce *PML* transcription through activation of the Janus kinase/signal transducer and activator of transcription (JAK/STAT) pathway [103, 104, 106]. Tumor necrosis factor alpha (TNFα) also activates *PML* transcription by promoting STAT1-dependent transactivation of the *PML* promoter [107, 108]. Moreover, interleukin 6 (IL-6) enhances *PML* transcription via NF-κB and JAK-STAT pathways [109]. In summary, *PML* transcription is tightly regulated by various cytokines.

In response to K-Ras-induced cellular senescence, p53 and its homolog p73 activate *PML* transcription, but this activation can be attenuated by Akt/PKB [110, 111]. Furthermore, β-catenin and plakoglobin are capable of activating the PML promoter in a LEF/TCF-independent manner in p53-negative KTCTL60 renal carcinoma cells [72].

In addition to transcriptional regulation, *PML* mRNA translation can also be regulated. In rodent cells, oncogenic K-Ras activates *Pml* mRNA translation in an mTOR- and eIF4E-dependent manner, presumably by targeting the *Pml* 5′-untranslated region of its mRNA [112]. We have recently demonstrated that the 5′-UTR of the human *PML* mRNA harbors an internal ribosome entrance site (*IRES*) that can be activated in response to TNFα. This *IRES* is conserved in most mammals except mouse [113].

### Post-translational regulation

In most cancers, PML protein level is down-regulated. However, the *PML* transcript level is usually comparable between normal and cancerous tissue [26]. These observations suggest that PML protein abundance is controlled post-transcriptionally. PML protein abundance and its functions are regulated by multiple post-translational modifications (PTMs), including ubiquitination, SUMOylation, phosphorylation, acetylation and peptidyl-prolyl isomerization [114, 115] (Table 1). Recent evidence indicates that there is crosstalk among these PTMs, which adds a complex layer of regulation to the control of the PML protein expression/function [116].

**Table 1:** PML post-translational modifications, regulators and effects of these modifications on PML protein levels

Regulators	Effect on PML	Target region/sites	References
KLHL20 (MLN4924), SIAH, E6AP, UHRF1	Down-regulates PML protein abundance by promoting ubiquitination		[120–124]
RNF4		SUMO-dependent	[118, 119]
KLHL39, USP11	Up-regulates PML protein by blocking ubiquitination		[125, 129]
MageA, HDAC7, PIAS1, SIRT1	Regulates PML SUMOylation	K65, K160, K490	[132–134]
		K490	[116, 131]
p300	Acetylation	K487, K515	[146]
SIRT1, SIRT5	De-acetylation, increase K490SUMOylation	K487	[9, 116]
Chk2	Phosphorylation	S117	[45]
CDK1/2		S518	[122]
ERK2		S403, S505	[142]
CK2 (Emodin)		S565^a^	[143]
BMK1 (XMD9-92)		S8, S38	[144]
HIPK2		S403, T409	[145]
SCP1, SCP2 and SCP3	De-phosphorylation	S518	[128]
Pin1	Isomerization	pS518-P519	[122, 141, 142]

PML protein is controlled by several post-translational modifications, including ubquitination, SUMOylation, acetylation, phosphorylation and isomerization

^a^ Annotated as S517 in reference due to different PML isoform

Inhibition of the proteasome pathway restores PML protein expression in select cancer cell lines [26, 117]. This observation suggested the possibility that abnormal ubiquitination and subsequent degradation of PML in cancer cells was involved. This prompted a search for the relevant E3 ligases targeting the PML protein. So far, at least seven E3 ligases have been identified that can ubiquitinate PML including RNF4, UHRF1, E6AP, KLHL1, KLHL20 and SIAH1/2 [118–124]. Interestingly, KLHL39 (kelch-like family member 39) interacts with PML and disrupts the binding of KLHL20 to PML and blocks KLHL20-mediated ubiquitination of PML [125]. We have previously shown that the peptidyl-prolyl cis-trans isomerase Pin1 binds phosphorylated PML at multiple sites that include S403 and S518 and promotes its degradation in triple-negative MDA-MB-231 breast cancer cells [126]. Additionally, AT-RA promotes Pin1 degradation and potently inhibits human triple-negative breast (TNB) cancer cell growth and tumor growth in TNB cancer animal models [127]. Moreover, the phosphatases SCP1, SCP2 and SCP3 dephosphorylate PML at S518, thereby blocking Pin1- and CDK2-dependent PML ubiquitination as well as KLHL20-mediated degradation [128]. By contrast, USP11 promotes deubiquitination and stabilization of PML [129].

PML is subject to SUMO1 monosumoylation on K490 and SUMO2/3 polysumoylation on K65 and K160 [118, 119]. The E3 ubiquitin ligase, RNF4, binds polysumoylated PML through its SIMs and promotes SUMOylation-dependent ubiquitination [118]. Interestingly, depletion of SUMO-3 reduces the number and size of PML-NBs [130]. SUMOylation of PML facilitates the recruitment of SIM-containing partner proteins to PML-NBs through their SIMs [21, 68]. RanBP2, SIRT1, HDAC7 and PIAS1 have been shown to promote PML SUMOylaiton, while MageA, a subfamily of the melanoma antigen genes, attenuates PML SUMOylation [116, 131–135]. Arsenic trioxide (ATO) is cytotoxic and ATO-mediated degradation of the PML-RARα fusion protein contributes to its therapeutic effect for APL patients [126, 136–138]. This process requires direct binding of ATO to PML protein [139] and depends on SUMOylation-dependent, ubiquitin-mediated degradation by RNF4 [118, 119].

Phosphorylation of PML can also modulate PML protein stability. In response to growth factors, IGF-1 or EGF, hypoxia, ERK2 or CDK1/2 phosphorylation of PML is enhanced which in turn promotes the interaction between phospho-PML and Pin1 [134, 140]. This interaction facilitates Pin1-mediated protein isomerization [122, 141, 142] followed by ubiquitination-mediated protein degradation. By contrast, high doses of H_2_O_2_ disrupt the PML and Pin1 interaction, thereby stabilizing PML [134, 140]. The CK2 kinase phosphorylates PML S565 and promotes PIAS1-mediated degradation of PML, although the identity of the putative ubquitin E3 ligase is unknown [133, 143]. Similarly, the Big MAP Kinase 1 (BMK1) down-regulates PML protein levels by phosphorylating PML at S403 and T409 and promoting its degradation, thereby disrupting the interaction between PML and Mdm2 and suppressing p53 activity [44, 144]. Unlike CK2, CDK1/2 or BMK1, DNA damage-activated HIPK2 promotes PML phosphorylation at S8 and S38, resulting in stabilization of PML [145].

PML is also subjected to acetylation at K487 and K505 by the protein acetyltransferase p300 [146]. Through screening all 18 known HDACs, we demonstrated that SIRT1- and SIRT5-mediate deacetylaiton of PML at K487 which is indispensible for H_2_O_2_-induced accumulation of nuclear PML and NBs and cell death in HeLa cells [116]. Furthermore, nuclear localization of PML is essential for H_2_O_2_-induced cell death [116].

Accumulating evidence indicates that crosstalk between the PTMs controls PML function. For example, the interaction between the ubiquitin E3 ligase, RNF4, and PML ubiquitination requires PML SUMOylaiton by Sumo2/3 [130]. Phosphorylation of PML protein by CDK1/2 or ERK2 is essential for Pin1 binding and Pin1-mediated protein isomerization [122, 141, 142]. CK2-mediated phosphorylation promotes proteasome-and ubiquitination-mediated degradation of PML [143] and the deacetylase SIRT1 promotes PML sumoylation and increases PML and PML NB abundance [131]. Lastly, we demonstrated that acetylation at K487 and sumoylation at K490 in PML are mutually exclusive, suggesting a negative crosstalk between these two modification [116].

## Mechanisms underlying nucleocytoplasmic shuttling of PML

All nuclear PML isoforms harbor an NLS. Disruption of the NLS by mutation at K487 results in accumulation of PML in the cytoplasm [9, 116, 147]. In addition, the longest isoform, PMLI, also contains a C-terminal putative NES (Fig. 2). An early study suggested that this NES is functional, but inefficient [148]. Currently, the mechanism by which the C-terminal NES regulates nucleocytoplasmic trafficking of PML1 and how the activity of the NES is regulated remain unknown.

In most studies, PML is localized both in the nucleus and cytoplasm. This can involve active re-distribution of PML. For example, in response to high doses of H_2_O_2_, SIRT1 and PML move from the cytoplasm to the nucleus and promote cell death in HeLa cells [116]. The HDAC catalytic activity of SIRT1 is essential for this H_2_O_2_-induced accumulation of nuclear PML. Because SIRT1 promotes deacetylation of PML at K487, a residue lying in the center of NLS, acetylation of K487 may influence PML nuclear localization by blocking recognition of the NLS by importins.

Recently, we discovered that oxidative stress and antioxidants control the subcellular distribution of PML. The antioxidant sulforaphane (SFN) is a potent inducer of cytoprotective genes [149]. The precursor of SFN, glucoraphanin, is abundant in cruciferous vegetables with its highest concentration found in broccoli [150]. Recent studies indicate that SFN induces apoptosis in cancer cells, inhibits cancer cell proliferation [151] and suppresses tumorigenesis in various mouse models of cancer [152]. We have recently demonstrated that PML is essential for SFN-mediated inhibition of capillary tube formation and migration of endothelial cells [147]. Notably, SFN induces an accumulation of cytoplasmic PML and a reduction in nuclear PML, although the underlying mechanism has not been elucidated. The role of PML nucleocytoplasmic trafficking in cellular activity remains an intriguing issue to address.

## Conclusion and perspective

One key direction for future study will be the role of PML in epigenetics and chromatin organization. Many histone modifying enzymes and enzymatic components of chromatin remodeling complexes interact with PML. For example, protein acetyltransferase (p300), deacetylase (HDAC1, HDAC2, HDAC3, HDAC7, SIRT1 and SIRT5) [65, 70, 116, 153, 154], methyltransferases (SETDB1 and SUV39H1) [155, 156], component of polycomb repressive complex (EZH2) [157], and epigenetic regulator UHRF1 physically associate with PML [120]. However, little is known about whether PML controls the activity of these chromatin regulators. Understanding the epigenetic regulation by PML is a pivotal step toward elucidating the mechanism of tumor suppression by PML and is reactivating PML in cancer cells. Currently, γ-irradiation and chemical therapies IFN and IL6, have been shown to stimulate accumulation of PML protein [49, 103, 104, 109]. PML protein abundance is also regulated by synthetic molecules, including MLN4924 (target KLHL20), emodin (target CK2), XMD8-92 (target BMK1) and TSA (HDAC inhibitor), as well as other nature compounds, such as H_2_O_2_, EGF, SFN, MG132, As_2_O_3_ and DNA damage regents [21, 115, 147, 158]. It will be informative to see whether combinatorial treatment with these reagents enhances potency of their anti-cancer activity by synergistically increasing PML protein accumulation.

In addition to its anti-proliferative and pro-apoptotic activity in tumor cells, PML can also influence the tumor microenvironment. PML inhibits neoangiogenesis, in part, by repressing translation of hypoxia-inducible factor 1 alpha (HIF-1α) through inhibition of mammalian target of rapamycin (mTOR) [159]. PML is essential for the TNFα- and IFNα-mediated inhibition of angiogenesis in ECs through repression of integrin beta1 (ITGB1) expression [108]. The anti-oxidant sulforaphane (SFN)-mediated anti-angiogenesis effects also requires PML protein [147]. Taken together, these findings suggest the suppressive function of PML in angiogenesis.

Finally, studies from Pandolfi’s group suggested that PML has an oncogenic function in chronic myeloid leukemia due to its importance in the maintenance of hematopoietic stem cells [160]. The same group also reported that PML is overexpressed in TNB cancer patients and suggested that PML is an oncoprotein in TNB [161]. In *Pml*^−*/*−^ mice, the neural progenitor cells are increased and the transition between two progenitor types, radial glial cells and basal progenitors, is disrupted [57]. These data suggest a role for PML in brain development. Interestingly, the number and size of PML NBs are significantly increased during glioblastoma stem cell differentiation [162]. However, it was recently demonstrated that Notch/Hey1 transcriptionally represses the expression of the PML deubiquitinase USP11, thereby down-regulating PML protein levels. In glioma patients, up-regulation of Hey1 correlates with down-regulation of USP11 and PML and with glioblastoma multiforme, a grade IV glioma [129]. Moreover, Hey1 overexpression or USP11 depletion blocks the anti-proliferation/migration/invasion effects of wild type PML but not a USP11-resistant PML mutant [129]. These observations suggest an inhibitory role of PML in the pathogenesis of GMB. Our lab recently demonstrated that *Pml* KO mice exhibited increased fatty acid oxidation in liver, which may contribute to a reduced incidence of Western diet-induced dysplastic hepatic nodules [163]. How PML may switch from a tumor suppressor in one tissue to an oncoprotein in another tissue is an outstanding question and warrants further investigation.

## Abbreviations

ALT: Alternative lengthening of telomeres
APL: Acute promyelocytic leukemia
BMK1: Big MAP kinase 1
HDACs: Histone deacetylases
HIPK2: Homeodomain-interacting protein kinase-2
INFs: Interferons
JAK/STAT: Janus kinase/signal transducer and activator of transcription
NAD+: Nicotinamide adenine dinucleotide
NBS1: Nijmegen breakage syndrome protein 1
NES: Nuclear export signal
NJEM: Non-homologous recombination repair
NLS: Nuclear localization sequence
Pin1: Peptidyl-prolyl cis/trans isomerase 1
PML: Promyelocytic leukemia protein
PML-NBs: Promyelocytic leukemia protein-nuclear bodies
pp1a: Protein phosphatase 1α
PP2a: Protein phosphatase 2α
PTEN: Phosphatase and tensin homolog
PTM: Post-translational modification
RARα: Retinoic acid receptor alpha
Rb: Retinoblastoma
RBCC domain, RING-finger: Two B-boxes and alpha-helical coiled-coil domain
RING domain: Really Interesting New Gene
SIM: SUMO interacting motif
SIRTs: Sirtuins
STAT3: Signal transducer and activator of transcription 3
SUMO: Small ubiquitin-like molecule
TGFβ: Transforming growth factor beta
TNB: Triple-negative breast
TRIM: Tripartite motif
TSA: Trichostatin A
UHRF1: Ubiquitin-like, containing PHD and RING finger domains 1
Ubc9: Ubiquitin-conjugating enzyme 9
